# Real-world evidence: How pragmatic are randomized controlled trials labeled as pragmatic?

**DOI:** 10.1186/s12916-018-1038-2

**Published:** 2018-04-03

**Authors:** Rafael Dal-Ré, Perrine Janiaud, John P. A. Ioannidis

**Affiliations:** 1 Epidemiology Unit, Health Research Institute-Fundación Jiménez Díaz University Hospital, Universidad Autónoma de Madrid, E-28040 Madrid, Spain; 2 Meta-Research Innovation Center at Stanford (METRICS), Stanford University, Stanford, CA 94305 USA; 3 Departments of Medicine, Health Research and Policy, Biomedical Data Science, Statistics, and Meta-Research Innovation Center at Stanford (METRICS), Stanford University, Stanford, CA 94305 USA

**Keywords:** Pragmatic trials, Explanatory trials, Real-world data, Effectiveness, Usual clinical practice, PRECIS-2, Editors

## Abstract

**Introduction:**

Pragmatic randomized controlled trials (RCTs) mimic usual clinical practice and they are critical to inform decision-making by patients, clinicians and policy-makers in real-world settings. Pragmatic RCTs assess effectiveness of available medicines, while explanatory RCTs assess efficacy of investigational medicines. Explanatory and pragmatic are the extremes of a continuum. This debate article seeks to evaluate and provide recommendation on how to characterize pragmatic RCTs in light of the current landscape of RCTs. It is supported by findings from a PubMed search conducted in August 2017, which retrieved 615 RCTs self-labeled in their titles as “pragmatic” or “naturalistic”. We focused on 89 of these trials that assessed medicines (drugs or biologics).

**Discussion:**

36% of these 89 trials were placebo-controlled, performed before licensing of the medicine, or done in a single-center. In our opinion, such RCTs overtly deviate from usual care and pragmatism. It follows, that the use of the term ‘pragmatic’ to describe them, conveys a misleading message to patients and clinicians. Furthermore, many other trials among the 615 coined as ‘pragmatic’ and assessing other types of intervention are plausibly not very pragmatic; however, this is impossible for a reader to tell without access to the full protocol and insider knowledge of the trial conduct. The degree of pragmatism should be evaluated by the trial investigators themselves using the PRECIS-2 tool, a tool that comprises 9 domains, each scored from 1 (very explanatory) to 5 (very pragmatic).

**Conclusions:**

To allow for a more appropriate characterization of the degree of pragmatism in clinical research, submissions of RCTs to funders, research ethics committees and to peer-reviewed journals should include a PRECIS-2 tool assessment done by the trial investigators. Clarity and accuracy on the extent to which a RCT is pragmatic will help understand how much it is relevant to real-world practice.

## Background

There is an increasing interest for obtaining evidence on the relative benefits and harms of interventions in real-world, every day circumstances. Real-world evidence can be obtained with observational data capturing routine care through comparative effectiveness research, such as, among others, large cohort studies, registry studies or retrospective studies on databases [1]; however, non-randomized information has major limitations and biases [2–4]. Because, pragmatic (or naturalistic) randomized controlled trials (RCTs) [5–7] can remove the biases due to lack of randomization and hopefully still provide evidence that closely captures routine care, they have become very attractive. The term “pragmatic” for RCTs was introduced half a century ago [8]. In contrast to “explanatory” RCTs that test hypotheses on whether the intervention causes an outcome of interest in ideal circumstances, “pragmatic” RCTs aim to provide information on the relative merits of real-world clinical alternatives in routine care. A critical aim of an explanatory RCT is to ensure internal validity (prevention of bias); conversely, a pragmatic RCT focuses on maximizing external validity (generalizability of the results to many real-world settings), but should try to preserve as much internal validity as possible.

When assessing new medicines (drugs or biologics) or new indications prior licensing, the typical RCT is highly explanatory (double-blind, placebo-controlled) [9]. Conversely, the typical paradigm of a real-world, comparative effectiveness medicines RCT is highly pragmatic, and compares the effectiveness of two commercially available medicines that are prescribed in routine care but have not been previously compared to each other [10]. Pragmatic medicine RCTs help to inform decisions by end users of information such as clinicians and patients, and by decision makers such as hospital, insurance and other policy makers. They are also used by industry in their price and reimbursement discussions with regulators. In an unsustainable health-cost environment, Health Technology Assessment agencies and Managed Care Organizations want to have real-world evidence on comparative effectiveness of available interventions in clinical practice to inform their decisions. To this end, pragmatic RCTs could play a critical role in defining which interventions should be recommended or prioritized.

Trials on regulated interventions (medicines, devices) have to be conducted following strict regulatory requirements that, almost invariably, prevent pre-approval trials to be pragmatic in nature. The need of medicines trials to be conducted following Good Clinical Practice guidelines implies that many trial features -from lengthy informed consent documents to providing the experimental medicine in non-commercial packages with the warning ‘investigational drug-for clinical trials use only’ - invariably disrupts any intention to mimic normal practice. Furthermore, complexity of phase 3 trials has increased over time, thus moving away from pragmatism: between 2001 and 2005 and 2010–2015, phase 3 trials have increased the mean number of planned visits, number of distinct procedures, and total number of procedures performed per trial by 27%, 59%, and 70%, respectively [11]. Conversely, a high degree of pragmatism is clearly an option for post-approval trials.

For trials on non-regulated interventions (e.g., surgery, physiotherapy, behavior), pragmatism is possible in both early and late development trials. However, it is very difficult for readers to appraise how close to usual clinical practice authors conducted their research in their own settings. To avoid speculating, we have based the argument of this paper on medicines trials only.

In this article we first set the scene by describing what are the features that define a pragmatic RCT and conducted a search on PubMed to know how prevalent is this type of RCTs. Second, we qualitatively discuss the retrieved papers assessing medicine pragmatic RCTs, and describe several trial features that prevent them from being labeled as pragmatic and propose how PRECIS-2 tool should be used by investigators to label trials as pragmatic in both submissions of protocols to research ethics committees and manuscripts to journals. Finally, we argue that both investigators and editors should use PRECIS-2 tool as a common, transparent and standardized method to appropriately label RCTs as pragmatic.

## Discussion

### Self-labeling randomized controlled trials as pragmatic

Currently, it is widely accepted that explanatory and pragmatic are the extremes of a continuum [12]. Many RCTs have both pragmatic and explanatory features. The issue is to know how pragmatic is a given trial to deserve to be named as such, especially when there are many investigators labeling their RCTs as pragmatic [13, 14]. As appreciation of the value of real-world evidence becomes more widespread, labeling a RCT as “pragmatic” is almost a badge of honor. Thus, we suspect that many RCTs currently self-labeled as pragmatic are not necessarily pragmatic enough.

A genuinely pragmatic RCT should fulfill at least two fundamental features. First, its conduct should resemble usual clinical practice. Second, the results should be applicable to multiple other settings, not only the one where the trial was conducted. Consequently, in principle, pragmatic RCTs of medicines should assess already marketed medicines (rather than those still in clinical development before licensing) and should be done in several sites providing care to heterogeneous populations. Some investigators [15] have argued that blinding can offer safeguards on internal validity in trials that otherwise have pragmatic intentions. However, pragmatism is heavily compromised by blinding. When RCTs compare different medicines head-to-head, using multiple placebos for blinding is a substantial deviation from usual clinical practice. Taking two masked medicines, one active and one placebo, is a very different patient experience than having to take only one medicine. Likewise, RCTs comparing a single active medicine versus a single placebo can hardly be pragmatic. The patient’s uncertainty about whether he/she will receive the active medicine or not would affect its motivation to participate and may also affect the therapeutic response compared with real life. Furthermore, the patient instead of going to his/her usual pharmacy to acquire (with or without co-payment or full-payment) the drug, would typically go to a designated pharmacy where he/she will be given (free of charge) assigned packages of drug or placebo. All this could produce the Hawthorne effect in many participants. Pragmatic trials should avoid blinding with the exception of using blinded assessors of outcomes, whenever possible [16].

### Illustration of the prevalence of pragmatic medicine randomized clinical trials

To assess the prevalence of pragmatic RCTs we conducted a PubMed search on August 8th, 2017 with the aim to capture articles using the terms ‘pragmatic’ or ‘naturalistic in their titles. Trials that only used the terms in their full text were not captured. We considered that trials that only used these terms in their full text are not profiting that prominently from their claims to pragmatism unlike trials using these terms in a highly visible manner in their titles. Abstracts were screened to identify medicines RCTs, i.e. trials assessing a medicine (drug, biologic) in at least one arm of the trial. We then examined the full text when any relevant information was not available in the abstract. Articles reporting results and those describing the protocol or design of the RCT were included. When having one or more articles for one RCT (e.g., one describing the protocol and another describing the results; or one describing the results and another conducting an economic analysis) we have counted only one trial. We found 615 RCTs, from 1977 to 2017, self-labeled as pragmatic in their titles. They have recently increased geometrically with 58% (354/615) of articles published in the last 3 years and a half (2014–2017). Only 89 (16%) of the 615 trials were on medicines. Five of these 89 trials addressed investigational medicines before licensing. Another 16 trials were single-center. Four trials used multiple placebos for blinding and another 14 trials used a single placebo. Overall, 32 of the 89 RCTs (36%) were pre-licensing, single-center, or placebo-controlled: in these circumstances, they cannot be appropriately termed ‘pragmatic’.

### Standardizing appropriate use of the term pragmatic: the PRECIS-2 tool

Jarow et al. [17] have recently described the FDA approach to real-world data and its use in regulatory decision making. Real-world evidence is defined by the data source and *degree* of pragmatism. The data source should be routine clinical care while the study design and conduct should have a *high degree* of pragmatism. Medicine RCTs can provide real-world evidence, if their execution reflect use in clinical practice [17]. We are dealing with a continuum and there are tools available to help investigators to assess the degree of pragmatism of their RCT [12, 18, 19]. The PRECIS-2 [12] is the most widely-known tool.

As of August 2017, the PRECIS-2 website [20] had 349 users, with an increased traffic due to the new NIH website devoted to pragmatic RCTs [21] (K. Loudon, personal communication). The PRECIS-2 tool comprises 9 scored domains (Table 1) addressing the most important RCT features. The scoring should be done prospectively, i.e. before a trial starts [12]. However, the PRECIS-2 tool can also retrospectively assess the degree of pragmatism of a RCT, after its conduct [22, 23]. For an outside reader of the trial report, this can be done reliably only if detailed and accurate information is published on all of the 9 domains. Unfortunately, sufficient details are almost never available. Interestingly, it has been shown that scores in the 9 domains of the PRECIS-2 tool could diverge between the theory (what investigators intended at the protocol development stage) and the practice (what actually happened once the RCT was implemented) [22]. Protocol or logistic modifications during the conduct of a trial can change its degree of pragmatism.

**Table 1:** PRECIS-2 tool nine domains and scoring method [12]

*Domain*	*Comment*
Eligibility	Who is selected to participate in the trial?
Recruitment	How are participants recruited into the trial?
Setting	Where is the trial being done?
Organisation	What experience and resources are needed to deliver the intervention?
Flexibility: delivery	How should the intervention be delivered?
Flexibility: adherence	What measure are in place to make sure participants adhere to the intervention?
Follow-up	How closely participants are followed-up?
Primary outcome	How relevant is to participants?
Primary analysis	To what extent are all data included?

*Score* (each domain): from 1 to 5 using a 5-point Likert scale

1 = very explanatory, 3 = equally pragmatic and explanatory, 5 = very pragmatic

### Clinical trials features that prevent trials from being pragmatic

As mentioned above, more than one in three (36%) trials on medicines that were labeled as pragmatic in their titles were placebo-controlled, pre-licensing, or single-center.

As clarified in the CONSORT extension for pragmatic trials [24], it is practically impossible for a pragmatic trial to be blinded: “Belief (or disbelief) in the intervention, extra enthusiasm and effort (or less), and optimism (or pessimism) in the self-assessment of outcomes may thus add to (or detract from) the effects of an intervention”- components which are part of the treatment effect in usual care. Blinding disrupts these components that differentiate effectiveness (the goal of pragmatic trials) from efficacy (the goal of explanatory trials). For placebo-controlled RCTs the ‘recruitment’, ‘flexibility-delivery’ and ‘flexibility-adherence’ domain scores in the PRECIS-2 tool (Table 1) may be 1 or close to the explanatory extreme. Use of placebos is an obvious deviation from the real world and only a few patients would choose to be recruited in a trial with such an artificial treatment experience where they don’t know what they are receiving for treatment. However, this has not prevented investigators labeling placebo-controlled trials as pragmatic [25–27].

Similarly, RCTs on medicines before they are licensed or assessing a new indication or dosage form could hardly be pragmatic, since they have to comply with clinical trials regulations that have no resemblance to their subsequent application in routine care. Such feature would affect the ‘recruitment’, ‘organisation’, ‘flexibility: delivery’, ‘flexibility: adherence’ and ‘follow-up’ PRECIS-2 domains resulting into scores of 1 or close to the explanatory extreme. However, both private [13] and public [28] sponsors are expanding the use of the term pragmatic to include RCTs conducted before licensing with open-label [13] and even double-blind, placebo-controlled designs [28].

Finally, acknowledging that this is a debatable point, single-center RCTs could almost never be pragmatic. It is almost impossible to have much certainty that the results obtained in one site are generalizable to other centers and settings; the ‘setting’ domain score will be 1 or close to the explanatory extreme. Yet, single-center RCTs are self-labeled and published as being pragmatic [29, 30].

Trials with features that defy pragmatism have been labeled as pragmatic in all types of journal, including major general medical journals such as *BMJ* [26, 31] and *Annals of Internal Medicine* [25]. These cases exemplify how the use of the term “pragmatic” needs better standardization.

As mentioned above, we focus here on pragmatic trials on medicines only. However, similar considerations can apply to trials of other types of intervention. The mere requirement to participate in a controlled clinical experiment already poses distance from everyday life experiences of interventions such as, for instance, cognitive behavior, diet, exercise or acupuncture. Some of these trials are probably not genuinely pragmatic, but it is difficult to judge without in-depth knowledge of their exact conduct and context. However, many trials self-labeled as pragmatic and conducted with interventions such as the ones mentioned above could have been truly pragmatic as opposed to the ones conducted with regulated interventions (i.e., medicines and devices). There are fewer impediments to achieve high degree of pragmatism for trials of non-regulated interventions and these trials can closely mimic real-world approaches to recruitment, flexibility (adherence), follow-up and other important aspects of pragmatism without any regulatory obligations.

### Publication of protocols and results of pragmatic randomized clinical trials

Ideally, the scores of all 9 domains of a pragmatic RCT should be on or close to the pragmatic extreme (scores ≥4), in order for this trial to be labeled as pragmatic. However, it is suggested that trials with scores ≥4 in 4–5 domains could be labeled as pragmatic provided the scores of the remaining domains are 3. Several trials fulfill this requirement. Some examples [32–34] of retrospectively assessed RCTs by authors are posted on the PRECIS-2 homepage [20]. Conversely, in the PRECIS-2 homepage [20], there are also trials with several domain scores close to the pragmatic extreme, but also one or more domain scores close to the explanatory extreme [35–37]. Calling these latter trials ‘pragmatic’ can be misleading. However, there is one type of exception: highly pragmatic trials where the intervention is how care is organized (and hence the ‘organisation’ domain score will be in the explanatory extreme [12]), could be labeled as pragmatic since this is just an explicit feature of the intervention.

The assessment of pragmatism by PRECIS-2 tool (i.e., the score obtained in each of the 9 domains and reasons supporting those scores) should be publicly disclosed. When submitting the manuscript of an RCT labeled as pragmatic, authors should submit their PRECIS-2 tool assessment as supplemental information, allowing for reviewers and editors to appraise the degree of pragmatism of the RCT. The assessment should be honest enough to provide information not commonly reported in manuscripts, such as, for instance, the nature and extent of participants’ information sheet. This type of information should be provided to support adjudicated scores. Otherwise, currently a reader cannot reliably appraise a trial on the degree of pragmatism without insider information.

The final PRECIS-2 tool assessment agreed between authors and journal editor should be published to inform readers of the reasons supporting the use of ‘pragmatic’ to describe the RCT and, consequently, whether the trial is gathering real-world evidence. If there are no sound reasons to label the RCT as pragmatic, the authors should avoid using the terms real-world evidence, effectiveness and usual clinical practice when referring to the design, conduct and results obtained.

We also propose a wider adoption of the PRECIS-2 tool at the protocol development and grant application stages. The scores of the 9 domains (and the reasons supporting them) should be included as part of the trial protocol to inform research ethics committees on the appropriateness of using the term ‘pragmatic’. Similarly, it should be included in the published protocols of trials that claim to be pragmatic. There are already several examples of published trial protocols that include the scores of the 9 domains [38–40]. Finally, the 9 domain scores should be included on clinical trials registries –such as, for instance, clinicaltrials.gov– as part of the trial description.

## Conclusions

Given the increasing value of real-world evidence, it is important to use the term “pragmatic” judiciously. As mentioned above, a homepage is available with information on how to use the PRECIS-2 tool, a toolkit, examples, podcasts and webinars [20]. Although the PRECIS-2 tool has shown to have good interrater reliability and reasonable discriminant validity [41], its use is not free of issues [42]. We should expect that future enhancements on this tool should be provided mainly by PRECIS-2 authors. Nevertheless, until a better tool is developed, its use in the editorial process should help investigators to better label RCTs as pragmatic. In the current situation, non-standardized subjective and non-transparent assessments can result in incorrectly labelling many RCTs as pragmatic. We may also introduce the PRECIS-2 tool scores in the CONSORT guidelines [24].

We do believe that journals, research ethics committees, Health Technology Assessment agencies, regulators, and funders may help ensure that the use of the term ‘pragmatic’ actually conveys the correct message of describing a RCT or a protocol thereof with a high degree of pragmatism. Appropriate use of the term will allow us to know how much real-world evidence we really have available from RCTs.

## Abbreviations

RCT (s): randomized controlled trial (s)
